# Patchless Multi-Stage Transfer Learning for Improved Mammographic Breast Mass Classification

**DOI:** 10.3390/cancers14051280

**Published:** 2022-03-01

**Authors:** Gelan Ayana, Jinhyung Park, Se-woon Choe

**Affiliations:** 1 Department of Medical IT Convergence Engineering, Kumoh National Institute of Technology, Gumi 39253, Korea; gelan@kumoh.ac.kr (G.A.); 20150555@kumoh.ac.kr (J.P.); 2 Department of IT Convergence Engineering, Kumoh National Institute of Technology, Gumi 39253, Korea

**Keywords:** multi-stage transfer learning, patchless, mammogram, classification, cancer cell line

## Abstract

**Simple Summary:**

In this study, we propose a novel deep-learning method based on multi-stage transfer learning (MSTL) from ImageNet and cancer cell line image pre-trained models to classify mammographic masses as either benign or malignant. The proposed method alleviates the challenge of obtaining large amounts of labeled mammogram training data by utilizing a large number of cancer cell line microscopic images as an intermediate domain of learning between the natural domain (ImageNet) and medical domain (mammography). Moreover, our method does not utilize patch separation (to segment the region of interest before classification), which renders it computationally simple and fast compared to previous studies. The findings of this study are of crucial importance in the early diagnosis of breast cancer in young women with dense breasts because mammography does not provide reliable diagnosis in such cases.

**Abstract:**

Despite great achievements in classifying mammographic breast-mass images via deep-learning (DL), obtaining large amounts of training data and ensuring generalizations across different datasets with robust and well-optimized algorithms remain a challenge. ImageNet-based transfer learning (TL) and patch classifiers have been utilized to address these challenges. However, researchers have been unable to achieve the desired performance for DL to be used as a standalone tool. In this study, we propose a novel multi-stage TL from ImageNet and cancer cell line image pre-trained models to classify mammographic breast masses as either benign or malignant. We trained our model on three public datasets: Digital Database for Screening Mammography (DDSM), INbreast, and Mammographic Image Analysis Society (MIAS). In addition, a mixed dataset of the images from these three datasets was used to train the model. We obtained an average five-fold cross validation AUC of 1, 0.9994, 0.9993, and 0.9998 for DDSM, INbreast, MIAS, and mixed datasets, respectively. Moreover, the observed performance improvement using our method against the patch-based method was statistically significant, with a *p*-value of 0.0029. Furthermore, our patchless approach performed better than patch- and whole image-based methods, improving test accuracy by 8% (91.41% vs. 99.34%), tested on the INbreast dataset. The proposed method is of significant importance in solving the need for a large training dataset as well as reducing the computational burden in training and implementing the mammography-based deep-learning models for early diagnosis of breast cancer.

## 1. Introduction

Breast cancer is the most commonly diagnosed cancer, followed by lung cancer. With an estimated 2.3 million new cases, breast cancer accounted for 12% of the total new cancer cases globally in 2021, according to the World Health Organization [1]. Health institutions recommend early diagnosis with mammography, which is crucial for mitigating the mortality rate of breast cancer [2]. Population-wide mammography screening resulting in the earlier detection of tumors has decreased breast cancer mortality rate by 40%, as reported in different studies [2,3]. Nevertheless, a high recall (asking a woman to return for additional workup after a screening mammogram) rate, which is due to significant false-positive and false-negative rates along with non-uniformity in the availability of an expert reader, is a major concern in mammographic breast-cancer screening [4,5]. Breast-mass characterization, the most important finding in screening breast cancer, particularly for women with dense breasts and under the age of 40, is where mammography fails to perform satisfactorily and is susceptible to false-positive and false-negative results [6,7]. If a mass is not a simple cyst, additional imaging tests such as ultrasound may be required to determine whether it could be a cancer [7,8]. Regular mammograms and ultrasound may take time to detect a change in some masses; therefore, a biopsy may be required to check the patients [7,9]. These tests can result in delayed diagnosis, unnecessary procedures, and can affect both patient experience and overall cost [10,11]. Given the importance of mammography in breast-cancer screening, there is an obvious need for an improved algorithm that accurately discriminates between benign and malignant mammogram breast-mass images.

To address the need for improving mammography performance, several recent studies have applied deep learning and highlighted two key difficulties: obtaining large amounts of training data and ensuring the generalization of these methods across different datasets using a robust and well-optimized algorithm that performs satisfactorily [12,13,14,15,16,17,18]. Many recent studies have applied ImageNet-based transfer learning (TL) to address the issue of unavailability of large datasets and patch-based deep-learning methods as robust algorithms [19,20,21,22]. However, there are still limitations in terms of accuracy, sensitivity, and specificity of these deep-learning methods when compared to radiologists [23,24]. Most efforts in transfer learning have focused on applying the existing techniques that have been applied on natural images to mammography rather than devising new ones that are particularly suited to the domain [25,26,27,28]. Medical images have properties differing from those of natural images [29]. For instance, in natural images, the crucial features determining a class mostly occupy a larger portion of the image; however, the regions of interest in medical images are usually relatively small compared to the size of whole image. The existing off-the-shelf convolutional neural network (CNN) architectures have been developed for natural images and do not consider the above-mentioned characteristics. Therefore, previous studies have failed to understand how the transfer-learning architectures pre-trained with natural images could be enhanced for mammograms. Furthermore, the algorithms in previous works are computationally complex, in which patch separation is carried out to segment the region of interest before the classification of the tumor, which makes the model-training process computationally infeasible and time consuming [30,31].

In this study, we propose a novel deep-learning method based on multi-stage transfer learning (MSTL) from ImageNet and cancer cell line images pre-trained models to classify mammographic breast mass as either benign or malignant. The importance of transfer learning is in the case of scarcity of a large training dataset, which enables a model to better generalize for target data based on previous knowledge from other domain data. In our case, we are supposed to have millions of mammographic breast-mass training images in a dataset to be able to develop an algorithm that works well on previously unseen data. However, this is not achievable because collecting and labeling millions of mammogram images is expensive. Even if we manage the cost, there are ethical issues that arise from using the personal information of patients, making mammogram images collected by different organizations inaccessible. Moreover, there are limited organizations that are able to use such a big dataset to achieve their purpose because of the computational capacity needed for large datasets. Therefore, an alternative approach must be sought, and here is where the usage of cancer cell lines as intermediate transfer learning stage comes to play. Cancer cell line images are not expensive to generate in a large amount compared to other medical images (i.e., mammogram, ultrasound, magnetic resonance image (MRI)) and there is no issue of personal information that may arise, making it accessible compared to other medical images. On top of that, cancer cell line images share similar features as that of mammogram images, so that learned features from cancer cell line images improve the learning scheme in the target mammographic image dataset. The proposed method alleviates the challenge of obtaining large-sized labeled-mammogram training data by utilizing a large number of cancer cell line microscopic images as an intermediate domain of learning between the natural domain (ImageNet) and medical domain (mammography). Utilizing the cancer cell lines further improves the learning process in classifying mammographic images because mammographic images and microscopic images share more resembling features than natural images, which help in improving the transfer learning process. Moreover, our method does not utilize patch separation to segment the region of interest, which makes it computationally simpler and faster compared to the previous studies.

## 2. Literature Review

Mammography is not only a widely used frontline modality, but also an effective technique for breast-cancer diagnosis. Mammography plays a significant role in the early diagnosis of breast cancer, which helps doctors to administer proper medication to their patients such that death from breast cancer can be avoided [2,32]. Accurately distinguishing the type of mass at the early stage of breast cancer is one of the most important tasks that doctors carry out [6]. However, mammography is prone to incorrect diagnostic results, particularly in early-stage breast cancer detection, because it is challenging to characterize the breast masses at early stages [3]. This is where deep learning comes into play, wherein a CNN, well-trained on mammogram data, is utilized to assist radiologists in deciding the diagnostic outcomes. Various studies achieved a performance similar to, or better than, well-trained radiologists in classifying breast mass states properly [15,16,17]. However, these studies have concluded two important challenges that act as a bottleneck in the success of deep learning for the classification of mammographic breast masses. 

The first challenge is the absence of a robust deep-learning algorithm that performs better than radiologists while being feasible for deployment in clinical setups. To address this issue, researchers have attempted to develop appropriate deep learning algorithms for mammographic breast-mass classification. These works can be broadly categorized into two types: deep learning algorithms based on breast-mass patches, and patchless (whole image) classifiers. In patchless classifiers, end-to-end deep-learning models have been directly employed to classify the entire mammogram. The prominent approach in patchless (whole image) classifier methods is the use of multiscale convolution kernels. In [33], the authors have proposed a multiscale all-convolutional neural network to classify masses using the entire mammogram. Their approach involves context-feature extraction, followed by classification. It was observed by the authors that integrating multiple scale features significantly increased performance while speeding up the whole process of classification compared to traditional classifiers. Similarly, Xie et al. [34] proposed an end-to-end deep-learning model based on a multiscale approach for classifying mammographic mass images without patch separation. Their method requires only labeled mammogram images for training and classification. They achieved this by generating multiscale feature maps, which enabled the model to learn the global and local structures of the image for classification. They reported that utilizing a multiscale approach improves not only performance, but also the computational efficiency. However, designing a better performing deep-learning algorithm based on patch classifiers has been studied by few researchers. The general concept of a patch-based classifier for mammographic mass classification is that the suspected area of the tumor, called the region of interest (ROI), is searched from the mammogram via searching algorithms, and based on the threshold probability set, the high-probability area of the image, which is called a patch, is extracted from the whole image. Consequently, the classifier is trained using the selected image patch, and the status of the mass is determined. Many studies have proven that the patch-based classification of mammographic mass images improved classification performance compared to the whole image-based classification. However, patch-based classifiers are computationally infeasible because they are slow and require a large memory. Representative works using the patch-based approach include the work by Chougrad et al. [35] in which they used the preselected ROI patches to train a classifier and further used transfer learning as a means of increasing the performance. In their study, they were able to obtain the best result with InceptionV3 fine-tuned for two convolutional blocks. Furthermore, some studies explored the use of more patches extracted from the mammogram images as a means of improving the performance. Lotter et al. [19] generated two different patches from a mammogram image and fused the extracted patches to achieve improved performance compared to a single patch-based approach. In [36], the authors proposed a two-view mammogram classification algorithm in which the breast mass features of craniocaudal (CC) and mediolateral oblique (MLO) views were extracted as patches and then fused together to obtain features from the two views. By doing so, they were able to achieve the best performance compared to the single patch-based methods.

The second challenge is the requirement of a large training dataset to achieve a high-performance deep-learning algorithm. Obviously, it is expensive to acquire large medical images; therefore, most of the previous studies on mammographic-mass classification have utilized augmentation and transfer learning to overcome the challenge of finding large training datasets. In [37], a ConvNet pre-trained on ImageNet was used to train unregistered mammograms and classify masses. In their study, authors observed that fine tuning using layers from the ImageNet pre-trained model improved the performance compared to randomly initializing a model in classifying mammographic breast mass images. Similarly, in [38], Dhungel et al. proposed the use of a deep-learning classifier for breast-mass classification based on a network pre-trained with regression to hand-crafted feature values and fine-tuned based on the annotations of the breast-mass-classification dataset. Consequently, the authors demonstrated that hand-crafted feature values and CNN with pre-trained models achieved better results than hand-crafted feature values and CNN without pre-trained models. Furthermore, the authors in [39,40] applied transfer learning to extract tumor information from mammogram images using models pre-trained on the ImageNet dataset. They improved performance using transfer learning. Moreover, to tackle the challenge of acquiring large amounts of labeled mammogram data, learning from unlabeled data using a small amount of labeled data has been proposed in [41]. This is a graph-oriented semi-supervised learning method where unlabeled data are gradually labelled via computed weights. The authors achieved better performance by utilizing the features learned from the abundant-unlabeled datasets.

In recent years, the concept of multi-stage transfer learning has become popular in the field of computer vision, where a model pre-trained on subsequent related domains is transferred learning to improve the learning on the target task [42]. The effectiveness of multi-stage transfer learning has been proven in different areas of applications, including management [42] and machine-failure detection [43]. In medical applications, a few studies have been carried out on digital breast tomography [44], magnetic resonance imaging (MRI) [45], X-ray [46], and computed tomography (CT) [47] image classification. In [44], transfer learning from an ImageNet pre-trained model for the classification of breast-digitized screen-film mammogram images followed by transfer learning from a mammogram pre-trained model was carried out to classify digital breast tomosynthesis images. The authors reported improved performance compared to the conventional ImageNet-only pre-trained model. Similarly, multi-stage transfer learning has been reported to be better than conventional transfer learning in [45,46,47].

## 3. Materials and Methods

### 3.1. Datasets

#### 3.1.1. Cancer Cell Line Dataset

We used a BITS (Biomedical Imaging, Therapeutics, and Sensing Lab) microscopic cancer cell line image dataset. The cancer-cell-line microscopic images included HeLa (human cervical cancer cells), MCF-7 (human breast cancer cells), and NCI-H1299 (human lung cancer cells), which were utilized within 6 months of being acquired from the Korean Cell Line Bank (Seoul, Republic of Korea). The cell lines were cultured in high-glucose Dulbecco’s modified Eagle medium containing 10% fetal bovine serum and 1% penicillin streptomycin. The prepared cells were incubated at 37 °C in a humidified incubator with 5% CO_2_. Images were acquired using an inverted optical microscope (IX73, Olympus, Japan). The cell lines were imaged every day for 7 days during the cell culture, and a total of 608 images were taken (247 images of HeLa, 149 images of MCF-7, and 212 images of NCI-H1299), with 6800 patches for each cell line, totaling 20,400 patches, before the augmentation.

#### 3.1.2. Mammography Dataset

In this study, we utilized three independent mammogram-image datasets, namely, the Digital Database for Screening Mammography (DDSM) [48,49], INbreast [50], and the Mammographic Image Analysis Society Digital Mammogram Database (MIAS) [51]; a mixed dataset, which is the union of the three datasets, was used to train and evaluate our patchless deep transfer learning model. Table 1 summarizes the datasets used in the study.

- Digital Database for Screening Mammography (DDSM): DDSM is a dataset used in many studies involving deep learning for mammographic breast cancer diagnosis. It is publicly available for researchers (accessible at http://www.eng.usf.edu/cvprg/mammography/database.html (accessed on 8 September 2021)). It is the largest publicly available database and has 2620 cases of mediolateral oblique (MLO) and craniocaudal (CC) views of both breasts, for a total of 10,480 images. The images include all types of findings, from normal images to images with benign and malignant lesions. It includes patient information, such as age, and has breast imaging reporting and data system (BI-RADS) annotations and breast-density annotations based on the American College of Radiology (ACR). The images are annotated as a pixel-level boundary of the findings.
- INbreast: The INbreast dataset is composed of full-field mammography images acquired between April 2008 and July 2010 from the Breast Center in CHSJ, Porto. INbreast is another popular publicly available dataset (accessible at https://biokeanos.com/source/INBreast (accessed on 8 September 2021)). with 410 images, including 115 cases, of which 90 cases are with MLO and CC views of each breast and 25 cases are from only one breast collected from women who underwent mastectomy. The dataset involves all types of findings. Information about the age of patients and family history as well as BI-RADS classification and ACR breast density annotations are provided. Biopsy results for BI-RADS 3, 4, 5, and 6 cases are also included. This dataset has strong annotation, including the labels of individual findings.
- The Mammographic Image Analysis Society’s digital mammogram database (MIAS): MIAS is the oldest mammographic image dataset that has been used to develop many deep-learning algorithms for breast cancer diagnosis (accessible at http://peipa.essex.ac.uk/info/mias.html (accessed on 8 September 2021)). MIAS is a dataset with 161 cases with MLO views only, constituting 322 digitized images. It involves all types of findings, including benign and malignant lesions as well as normal images. It possesses breast-density information that is not classified according to ACR standards. The annotation was performed in such a way that the center and radius of a circle around the area of interest are provided.
- Mixed dataset: The mixed dataset was formulated by mixing the three datasets (DDSM, INbreast, and MIAS) to investigate the robustness of the proposed method for datasets from different sources. The mixed dataset was formed in such a way that all the breast mass images from DDSM, INbreast, and MIAS datasets are added together to form a larger, diversified dataset. The benign images from the three datasets formed a benign mixed dataset whereas the malignant images from the three datasets formed a malignant mixed dataset.

#### 3.1.3. Dataset Size and Categories

The study cohort was prepared by extracting the breast-mass images from all three dataset sources to form a mammographic breast mass image sub-dataset for the three independent datasets. Consequently, 2188 cases from DDSM, 106 cases from INbreast, and 53 cases from MIAS were extracted. Subsequently, augmentation was carried out to increase the number of datasets for improved feature learning. This increases the dataset size to 13,128 images of DDSM, 7632 images of INbreast, and 3816 images of MIAS mammographic breast mass image datasets. Additionally, a mixed dataset, consisting of a union of the three datasets comprising 24,576 mammographic-breast-mass images was prepared to study the robustness of the proposed model across a mixed dataset of different sources. Finally, each dataset was categorized into a 6:2:2 ratio for training, validation, and testing, respectively, as shown in Table 2.

### 3.2. Pre-Processing

**Figure 1:**
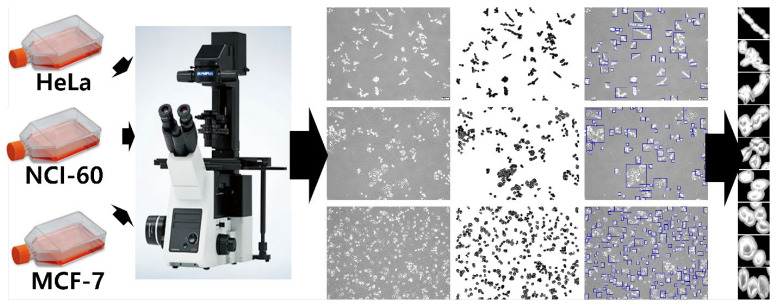
Cancer cell line image acquisition and pre-processing.

The pre-processing (see Figure 1) performed on the cancer cell line images includes adaptive thresholding to binarize the acquired image, followed by selecting the area of interest using OpenCV bounding box [52]. To prevent the outline of a small floating object other than the cell from being detected, an object with less than a certain number of pixels (100 × 100 pixels) is removed using the remove_small_objects function of Scikit-image. The selected cell region was extracted as an input image for learning and validation with a size of 300 × 300 pixels. By segmenting 608-cell bright-field images obtained through the microscope, 6800 images of each cell line were randomly chosen to form a total of 20, 400 datasets. The cancer-cell-line data were categorized using a 7:2:1 ratio for training, validation, and test sets (that is, 14,280 for training, 4080 for validation, and 2040 for test). The training data were further augmented (via rotation, width and height shift, and vertical flip (vertical flip is preferred over horizontal flip for microscopic images because microscopic images are inverted)) to increase the training dataset size to 28,560 images. 

**Figure 2:**
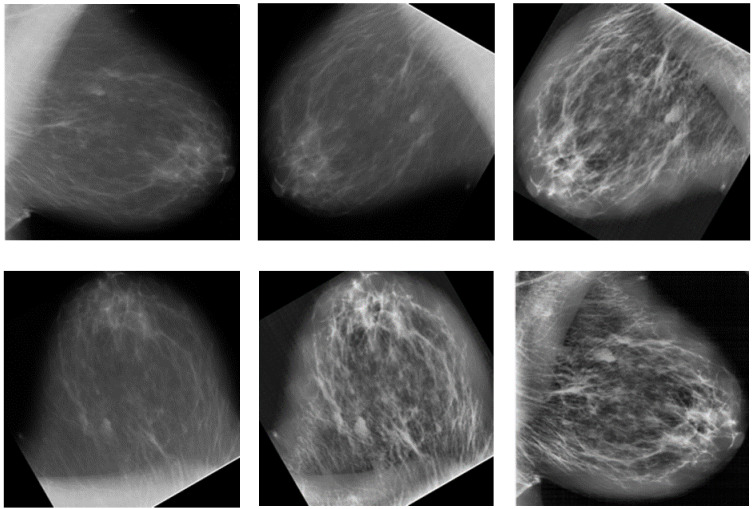
Different images formed after augmentation (Right MLO benign images from DDSM).

The mammogram images were augmented [53], including horizontal shift, vertical shift, horizontal flip, vertical flip, and random rotation augmentations, as shown in Figure 2. Furthermore, contrast-limited adaptive histogram equalization was performed on the images. The mammographic images were originally of different pixel sizes and were resized to a uniform size of 227 × 227 pixels.

### 3.3. The Deep-Learning Method

**Figure 3:**
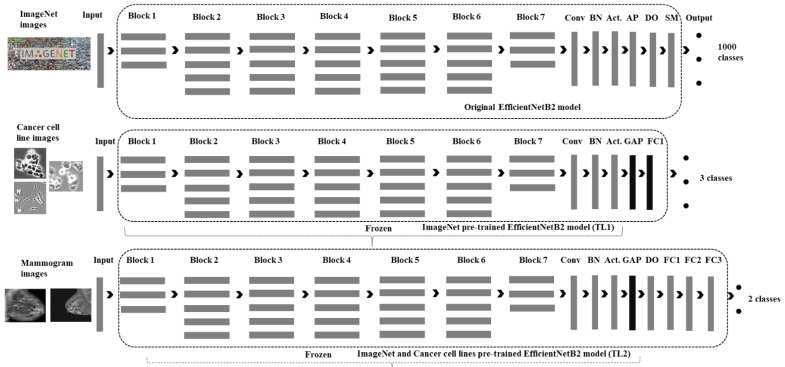
The proposed EfficientNetB2 based patchless multi-stage transfer-learning method for mammography breast mass image classification. FC: fully connected; TL: transfer learning; Conv: convolution; BN: batch normalization; Act.: activation; AP: average pooling; DO: drop out; SM: Softmax; GAP: global average pooling.

We developed a novel deep-learning method based on multi-stage transfer learning from ImageNet [54] and a pre-trained cancer cell line image model to classify mammographic breast masses as either benign or malignant, as shown in Figure 3. A multi-stage transfer learning process was designed in such a way that a model trained on the ImageNet dataset for classifying the natural images into 1000 classes is used as a pre-trained model to classify the cancer cell line images into three classes, namely, breast cancer, cervical cancer, and lung cancer, via transfer learning with a slight modification. Consequently, this model, pre-trained on cancer cell line images to classify them into three classes, was in turn used as a pre-trained model to classify mammography breast-mass images into two classes: benign and malignant.

We utilized a modified version of EfficientNetB2 [55] CNN architecture. In general, the EfficientNet models perform well in terms of both accuracy and efficiency, compared to existing CNNs, while lowering the parameter size and floating-point operations per second (FLOPS) by an order of magnitude. For instance, compared with the widely used ResNet, EfficientNet improves the accuracy of ImageNet by 6.3% using a similar FLOPS. EfficientNet performs better because it uniformly scales every dimension with a constant set of scaling coefficients in contrast to conventional approaches, which promptly scale network dimensions including width, depth, and resolution [55]. EfficientNet models outperform the other models with state-of-the-art accuracy and up to ten times better efficiency (smaller and faster) [55].

We modified the EfficientNetB2 network to enable it to provide a vector of binary labels indicating the possible type of mammography breast mass images. To do so, we used the ImageNet pre-trained EfficientNetB2 model and modified it to use it as a pre-trained model to classify the cancer cell line images into three classes. To modify the model, we changed the input layer for the appropriate cancer cell line image size and resolution. Subsequently, the average pooling on the original EfficientNetB2 model was replaced with global average pooling, and one additional fully connected layer was added with a Softmax output layer as shown in Figure 3. This model, pre-trained on the cancer cell line images, was subsequently used as a pre-trained model with a modification to classify mammography breast mass images as benign or malignant. The modifications include the application of a dropout along with the addition of three dense layers and a Softmax, as shown in Table 3. The final output is the probability distribution of the two classes.

### 3.4. Implementation Details

We trained EfficientNetB2 model with pre-trained weights from ImageNet as well as cancer cell line images. A careful study of the hyper-parameters effect resulted in the best choice of model parameters. In order to do so, we picked three state-of-the-art CNN architectures, including ResNet50, InceptionV3, and EfficientNetB2 architectures and three optimizers, including stochastic gradient descent (SGD), Adam, and Adagrad. Using the same parameters, such as batch size, learning rate, epoch number, and other parameters, we carried out a preliminary study to pick the best model and optimizer combination. Consequently, the combination of the EfficientNetB2 model and the Adagrad optimizer resulted in a relatively better result on all three datasets. Following this, we carried out optimization for the EfficientNetB2–Adagrad combination by varying parameters such as batch size, learning rate, epoch number, optimizers, and others.

Along these lines, we report the parameter values that achieved the best results. Our EfficiebtNetB2 model was trained for 150 epochs using Adagrad with a momentum of 0.96. We set the dropout rate to 0.6 and the initial learning rate to 0.001, which subsequently decayed exponentially. 

After fixing the different parameters of our model, we carried out improvement involving arrangement of the datasets. We have employed two dataset categorization approaches in which we have categorized our datasets in to 7:2:1 and 6:2:2 ratios for training, validation and tests. Based on the results from the studies, we decided to use the 6:2:2 ratio as it involves greater size of the test dataset and the results were comparable. Then, we performed the same procedure to generate the results reported in this paper using the nested 5-fold cross-validation for each of the training, validation, and test partitions.

The deep-learning model was implemented on RTX 3090 GPUs. The model was trained for 150 epochs at each TL stage, which was selected after careful study using early stopping and a fixed epoch of 150, as shown in Table 4. We ran our model using early stop with two patience values, 5 and 10, with respect to loss (that is, whether the loss is constant or decreasing for five consecutive iterations). With both patience values of 5 and 10, the model found its optimal value of epochs to be 150. However, when the model runs for both early stop and a fixed epoch of 150, the loss is relatively low for the fixed epoch; consequently, we chose a fixed epoch of 150. The training batch size was set to 16.

### 3.5. Experimental Settings

#### 3.5.1. Patchless Multi-Stage Transfer Learning Evaluation Experimental Settings

The proposed patchless multi-stage transfer-learning method was employed using three independent datasets, and performance metrics based on 5-fold cross validation were recorded. The EfficientNetB2 architecture with the Adagrad optimizer was used to evaluate the performance of the proposed method. The implementation details are as discussed in the previous section (see Section 3.4).

#### 3.5.2. The Dataset and Algorithm Wise Robustness Analysis Settings

Two evaluations were performed to determine the robustness of the proposed method for a dataset from different sources in terms of the acquisition device, type of image, and geographical location of the data sources. The evaluations were also performed to determine the algorithm-wise robustness of the proposed model over different architectures proposed by various researchers. Therefore, we mixed the datasets from the three sources, i.e., DDSM, INbreast, and MIAS, to form a mixed mammogram dataset. The implementation details of the proposed method on the mixed dataset were the same as those of the individual datasets. We categorized individual datasets into a 6:2:2 ratio for training, validation, and random testing. Following this, we mixed each training dataset from the three independent datasets to form a mixed training dataset. The same procedure as that of the mixed training dataset was followed to form the validation and test mixed datasets. This forms a dataset categorized as training, validation, and testing with a ratio of 6:2:2. The remainder of the processing was the same as that of the individual datasets.

Furthermore, the algorithm-wise robustness analysis of the proposed patchless multi-stage transfer learning model was performed using two additional CNN models, ResNet50 [56] and InceptionV3 [57], and two additional optimizers, Adam and SGD optimizers [58]. For both ResNet50 and InceptionV3 models, the same modification as that of EfficientNetB2 were employed, that is, a dropout along with the addition of two dense layers in place of the original model’s final layer and a sigmoid function in place of Softmax. The models were trained for 150 epochs with Adam and SGD with a momentum of 0.96, similarly to the EfficentNetB2 and Adagrad model. The same dropout rate of 0.6 and an initial learning rate of 0.001, which subsequently decayed exponentially, were used. The dataset settings were also the same to ensure unbiased comparison.

#### 3.5.3. Comparison of the Proposed Multi-Stage Transfer Learning (MSTL) Method against the Conventional Transfer Learning (CTL) Method Settings

Evaluation of our multi-stage transfer-learning method by comparing it with the conventional ImageNet-based transfer learning was performed by choosing the CNN model and optimizer reported to be the best-performing by previous papers [19,21,22]. Consequently, the ResNet50 architecture and Adam optimizer were utilized for comparison in this study. In the case of conventional transfer learning, the ImageNet-pre-trained ResNet50 model with Adam optimizer was used to classify mammographic breast mass images on the three datasets as well as on a mixed dataset. The ImageNet pre-trained ResNet50 model was modified in such a way that the average pooling layer changed into global average pooling and the Softmax layer changed into a sigmoid layer. The model was trained for 150 epochs using a training batch size of 16. All the weights were frozen during training, except for the last layer.

#### 3.5.4. Comparison against Patch and Whole Image Classifier Settings

Li Shen et al. proposed a deep-learning algorithm in which a model that classifies local image patches is pre-trained on a well-annotated dataset with ROI information [31]. The weight parameters of the patch classifier are then utilized to initialize the weight parameters of the entire image classifier, which can be further fine-tuned using datasets without ROI annotations. In [30], the authors used a DDSM dataset to construct the patch and whole image classifier, and then employed it on the INbreast dataset as the whole image classifier. Following the same procedure, we deployed a patch and whole-image classifier using ResNet50 architecture and Adam optimizer as in [31] and compared its performance against our proposed patchless method using the same architecture and optimizer, that is, ResNet50 and Adam. The DDSM dataset was utilized for training, and the INbreast dataset was utilized for testing, as in [31].

#### 3.5.5. Evaluation and Statistical Analysis

To evaluate the proposed patchless MSTL method, performance analysis in terms of the area under the ROC curve (AUC) [59], specificity, sensitivity, and F1 measure were additionally employed to test accuracy [60]. These performance metrics were evaluated by averaging over five-fold, nested cross-validation results [61]. The five-fold cross-validation shuffles data randomly and then divides it into five equally sized subsets that help to combat the risk of having a model that works well on training data but fails on the data it has never seen before. We also performed Student’s *t*-test [62] to evaluate the significance of the improvement from using our method against the conventional ImageNet-based transfer learning, which is widely used in different studies.

## 4. Results

We evaluated the performance of the proposed method by training and testing it with image datasets from three sources, namely, DDSM, INbreast, and MIAS datasets, as well as a mixed dataset from the three sources. The results, which were calculated using F1 measure, AUC, test accuracy, sensitivity, and specificity, are summarized in Table 5. Generally, the proposed model performed well on the DDSM dataset, as expected, because of the large size of the training dataset. Moreover, the model’s higher performance was recorded from the mixed dataset following the DDSM dataset. The reason for this may be that the model leverages the richness of information using datasets from different sources to learn more features. The higher performance of the mixed dataset implies the robustness of the proposed model across different datasets from different sources.

### 4.1. Results on DDSM

**Figure 4:**
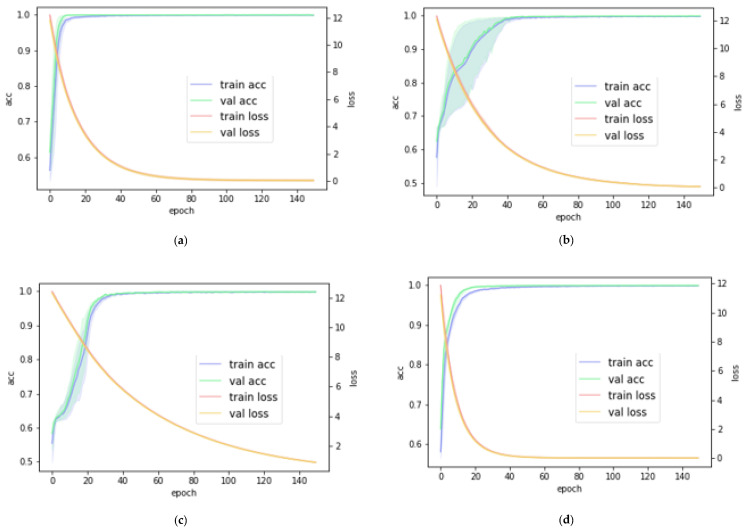
The learning curves of the proposed patchless deep-learning method for mammography breast-mass image classification on the (**a**) DDSM dataset; (**b**) INbreast dataset; (**c**) MIAS dataset; and (**d**) mixed dataset. train acc: training accuracy; val acc: validation accuracy; train loss: training loss; val loss: validation loss.

We found that our proposed method effectively distinguished between malignant and benign breast-mass images in the DDSM dataset with an average 5-fold cross-validation F1 score of 1, AUC of 1, test accuracy of 1, sensitivity of 1, and specificity of 1. The proposed method misclassified no image among the test images involving all the cases. Figure 4a shows the learning curve for the classification of benign and malignant breast- mass images for the DDSM dataset.

### 4.2. Results on INbreast

We found that our proposed method effectively distinguished between malignant and benign breast-mass images in the INbreast dataset with an average 5-fold cross validation F1 score of 0.9995, AUC of 0.9994, test accuracy of 0.9993, sensitivity of 0.9996, and specificity of 0.9992. Figure 4b shows the learning curve for the classification of benign and malignant breast mass images.

### 4.3. Results on MIAS

We found that our proposed method effectively distinguished between malignant and benign breast mass images in the MIAS dataset with an average 5-fold cross validation of F1 score of 0.9989, AUC of 0.9993, test accuracy of 0.9992, sensitivity of 0.9987, and specificity of 1. Figure 4c shows the learning curve for the classification of benign and malignant breast mass images.

### 4.4. Robustness Analysis Using Mixed Dataset

The performance of deep-learning models must be consistent and reproducible over mammograms obtained from different sources with mammography devices from various vendors to be used in clinical settings. A close evaluation of the performance of deep-learning algorithms across various devices is necessary because different devices from different vendors follow their own systems in generating the images for usage. This has a significant effect on the generalization of CNN models for mammography images from different sources. To evaluate the robustness of the proposed method over different dataset sources, we also trained and tested the proposed model on a mixed dataset from the three dataset sources, that is, DDSM, INbreast, and MIAS. We found that our proposed method effectively distinguished between malignant and benign breast mass images in the mixed dataset with an average 5-fold cross validation F1 score of 0.9998, AUC of 0.9998, test accuracy of 0.9998, sensitivity of 1, and specificity of 0.9997. Figure 4d shows the learning curve for the classification of benign and malignant breast mass images.

### 4.5. Robustness Analysis Using Other CNN Architectures and Optimizers

We have also employed our multi-stage transfer-learning method using different models and optimizers, as shown in Table 6, to examine the robustness and possible extension of our method to other state-of-the-art deep-learning algorithms using additional ResNet50 and InceptionV3 models as well as Adam and SGD optimizers. By doing so, we observed that our model performed consistently across other CNN models and optimizers, providing results close to those obtained using the EfficientNetB2–Adagrad combination. Notably, the EfficientNetB2–Adagrad combination showed the highest test accuracy across all datasets, with a test accuracy of 1 for DDSM, 0.9995 for INbreast, 0.9989 for MIAS, and 0.9998 for mixed datasets. However, for the DDSM dataset, even the ResNet50–Adagrad combination achieved the same test accuracy of 1 compared to the EfficientNetB2–Adagrad combination. This implies that the proposed method reproduces higher results, irrespective of utilizing different model and optimizer combinations.

### 4.6. Comparison of the Proposed Multi-Stage Transfer Learning (MSTL) Method with Conventional Transfer Learning (CTL)

We calculated the *p*-value of the t-test to determine the significance of the improvement due to the usage of cancer cell images in the second stage of our MSTL to compare it with the CTL (Table 7). We implemented conventional transfer learning by using the ResNet50 CNN and Adam optimizer based on the highest performance reported for conventional transfer learning by previous popular works in mammography [19,31]. Note that the ResNet50–Adam combination is not the best-performing model in our case. However, to be able to use the best practice for the CTL as reported in previous works, we compare our method and the CTL method based on the ResNet50–Adam combination. We considered the average five-fold cross-validation test accuracy results of each dataset as a single entry to obtain the *p*-value. The resulting *p*-value was 0.044 (that is, a probability of 4.4% that the improvement in performance from using our method will be false), which is less than the standard 0.05 (that is, 5%) significance cut-off *p*-value. This shows that our MSTL achieved a significant improvement in classifying mammographic breast mass images on all the datasets that were used, compared to the CTL that was based on the non-best practice in our case. Furthermore, we compared the CTL best practice with our proposed best model to determine the statistical significance of the accuracy improvement achieved by using our best model. The resulting *p*-value was 0.00294 (that is, a probability of 0.294% that the improvement in performance from using our method will be false), which is far less than 0.05 (that is, 5%), the standard significance cut-off *p*-value. This shows that our MSTL made a significant performance improvement in classifying mammographic breast mass images on all the datasets that were used, compared to CTL. Moreover, the proposed patchless multi-stage transfer-learning method outperformed the patch classifier-based model in both cases (using the best practice for patch classifiers based on the ResNet50 and Adam optimizer, as well as using our best model based on EfficientNetB2 and Adagrad) on individual datasets. On the DDSM dataset, the ResNet50–Adam model-based patchless multi-stage TL method, with a test accuracy of 90.898%, achieved 5% more accuracy than the patch-based classifier, which demonstrated a test accuracy of 85.723%. Similarly, our best model based on EfficientNetB2–Adagrad, with a test accuracy of 100%, achieved 14% more accuracy than the best practice using a patch classifier, which demonstrated a test accuracy of 85.723%. The same was observed using the other datasets also. Furthermore, the proposed method was computationally feasible compared to the conventional transfer learning method, with a relatively smaller training time, as shown in Table 7. For the same architecture and optimizer (ResNet50 and Adam), the proposed method training time was 1.709594928 h while the training time for the conventional transfer learning was 1.833570639 h, averaged over the four datasets. Here, the network sizes are almost the same except for the conventional transfer learning; the last pooling layer of the original ResNet50 architecture has been replaced with a new global pooling layer followed by one fully connected layer, whereas in the multistage transfer learning, two additional fully connected layers and a drop-out layer were added to the original architecture. The training time was even smaller for the proposed method using EfficientNetB2 and Adagrad, with a training time of 1.561507118 h, averaged over the four datasets.

### 4.7. Comparison of the Proposed Method with Patch and Whole Image Classifier

We developed a patch and whole-image classifier based on [31] and compared its performance with that of our proposed patchless method. In doing so, we used the same model and optimizer as in [31], which is based on the ResNet50 CNN and Adam optimizer. Moreover, we trained our model on the DDSM dataset and tested it on the INbreast dataset following the setup in [31]. Consequently, the patch and whole image classifier achieved a 5-fold cross validation average test accuracy of 91.41%, whereas the proposed patchless method achieved a 5-fold cross validation average test accuracy of 99.34% on the INbreast dataset (see Table 8). This demonstrates that our patchless approach performs better than patch- and whole image-based methods. Moreover, the proposed method reduced computational complexity by converging faster than the patch- and whole-image classifier, resulting in a shorter training time. The training time for the proposed method was 1.734480832 h, whereas the training time for the patch-and whole image classifier was 2.043398834 as reported in Table 8. For the patch and whole-image method network, we utilized original ResNet50 architecture where the last pooling layer was replaced with a spatial pyramid pooling layer followed by a flattening layer and two fully connected layers. The proposed patchless multistage transfer learning method utilized the original ResNet50 model where the last pooling layer is replaced with a global pooling layer, followed by a drop-out and three fully connected layers.

## 5. Discussion

Failure to accurately identify the state of breast mass widely causes false positive and false negative findings in the early diagnosis of breast cancer using mammography, calling for unnecessary biopsy and diagnosis using other imaging modalities. To address this issue, we developed a patchless multi-stage transfer-learning model to accurately classify mammographic breast masses as benign or malignant. In our model, a well-established EfficientNetB2, pre-trained on the ImageNet dataset to classify natural images into 1000 classes, is used via transfer learning with the necessary modifications to classify cancer cell line images into three categories. This model, which was trained on both ImageNet and cancer cell line images, is used as a pre-trained model for classifying mammographic breast mass images as benign or malignant by fine-tuning it with modifications on the network. The proposed model was implemented using three publicly available mammography datasets: DDSM, INbreast, and MIAS, as well as a mixture of these three datasets. The proposed model, based on the EfficientNetB2 architecture and Adagrad optimizer, achieved AUCs of 1, 0.9994, 0.9993, and 0.9998 for DDSM, INbreast, MIAS, and mixed datasets, respectively. Moreover, the proposed patchless multi-stage transfer-learning method performed better than the models using conventional transfer-learning methods (that is, models only pre-trained on ImageNet before being utilized for classifying mammographic breast-mass images). The performance improvement of our model was statistically significant; our model provided a *p*-value of 0.00294 over the best-performing conventional ImageNet-based transfer learning model. Furthermore, our patchless approach performed better than the patch- and whole image-based method improving accuracy by 8% (91.41% vs. 99.34%), tested on the INbreast dataset.

Representative deep-learning studies related to the classification of mammographic breast mass using the datasets in our study are summarized in Table 9, which shows that our patchless multi-stage transfer-learning method outperforms most of the state-of-the-art methods implemented using the same dataset. Among the studies that used the DDSM dataset, Al-masni et al. [22] reported an AUC of 0.9645 and an accuracy of 96.33%; Al-antari et al. [21] reported a maximum accuracy of 97.5% among the different architectures they used; Chougrad et al. [35] reported an AUC of 0.98 and an accuracy of 97.35%; Lotter et al. [14] reported an AUC of 0.92; and our proposed method achieved an AUC of 1 and a test accuracy of 1. Among the studies carried out using the INbreast dataset, Al-antari et al. [21] reported a maximum accuracy of 95.32% among the different architectures used; Ribli et al. [63] reported an AUC of 0.95, Chougrad et al. [35] reported an AUC of 0.97 and an accuracy of 95.5%; Dhungel et al. [38] reported a maximum AUC of 0.91 and a maximum accuracy of 95%; whereas our proposed method achieved an AUC of 0.9994 and a test accuracy of 0.9993. Furthermore, among the studies performed using the MIAS dataset, Chougrad et al. [35] reported an accuracy of 98.23% and an AUC of 0.99, and Saraswathi and Srinivasan et al. [64] reported an accuracy of 94.7%, whereas our proposed method achieved an AUC of 0.9993 and a test accuracy of 0.9992. From these comparisons, it can be inferred that our proposed method outperforms state-of-the-art methods trained and tested using the same datasets, even though there are differences in the experimental settings of these previous studies and our study. 

Most of the popular works in the area of deep learning for mammographic breast cancer images involve patch classification, where finding the region of interest is performed using moving windows, and the patches made with these moving windows are used as input for training the deep-learning algorithms. Despite their success in improving performance, these algorithms are computationally complex and time-consuming. Our patchless deep-transfer learning algorithm achieved better performance than that of the patch and whole-image classifiers for mammographic breast cancer classification trained on the DDSM dataset and tested on the INbreast dataset. Our method achieved better performance, with a test accuracy of 99.34%, compared to that of the patch and whole-image classifier, with a test accuracy of 91.41% averaged over 5-fold cross validation. The main reason for this is the use of the cancer cell line image-pre-trained model as an intermediate transfer learning dataset in our case, rather than directly using an ImageNet only pre-trained model. The use of cancer cell line images enables the transfer learning model to acquire knowledge of features from microscopic images, which are more related to mammographic images than the natural images in the ImageNet dataset.

To evaluate the robustness of our patchless multi-stage transfer learning model, we mixed the three datasets from DDSM, INbreast, and MIAS and trained our model on this mixed dataset. The performance of our model for the mixed dataset was consistent with that of the individual datasets from the same source. This proves the robustness of our model for any dataset from different sources, such as those from different institutions or devices. This is an important finding because most deep-learning algorithms fail to perform consistently for mammogram datasets from different sources. Furthermore, we deployed our model using two additional CNN architectures (ResNet50 and InceptionV3) and two optimizers (Adam and SGD). The results suggest that our approach is robust across different CNN architectures and optimizers (see Table 6). 

**Figure 5:**
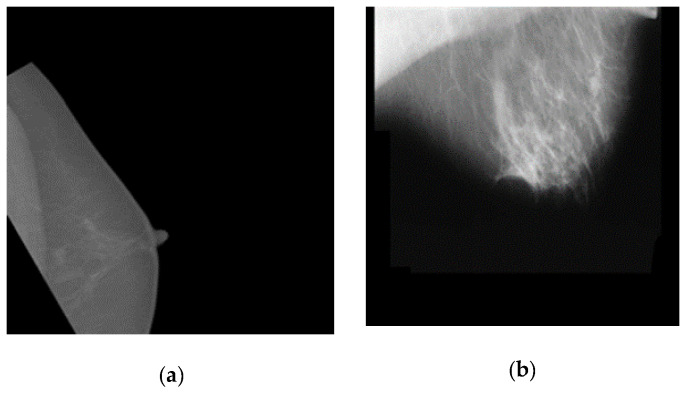
Examples of missing images from: (**a**) INbreast and (**b**) MIAS datasets.

The proposed model based on the EfficientNetB2 architecture and Adagrad optimizer did not miss any image in classifying the DDSM dataset. However, the model missed a few low-quality images from the INbreast and MIAS datasets. Figure 5 shows the images from the INbreast, MIAS, and mixed datasets that were missed by the proposed model. It can be easily deduced from these missed instances that these images were not properly acquired and were of low quality.

Undoubtedly, this work is of great importance for early breast-cancer diagnosis because of its primary focus on breast mass-based cancer discrimination, which is crucial for breast cancer screening in young women with dense breasts. The strengths of this work include the use of microscopic cancer-cell-line-image features related to mammographic images that serve as an intermediate domain between the natural image (ImageNet) and the target medical image (mammogram) domain training to achieve a high-performance deep-learning algorithm. The usage of cancer cell lines as an intermediate transfer learning stage helps the model to acquire more knowledge of the medical image domain because microscopic images and other medical images share more similar features than with natural images [65]. On top of this, the cancer cell line image dataset can be generated in a larger size compared to other medical images in terms of costs and ethical issues, which makes cancer cell line images the primary candidates for the intermediate transfer learning stage. The other advantage of this work is that it does not apply patch separation prior to training, which decreases the computational complexity and time required for training. This would be of great importance in the clinical application of deep-learning methods because it has the ability to be employed with reasonable resource allocation. In addition, deep learning based on the whole breast images would provide additional information that would help the model to learn more features other than the region of interest, in contrast to patch-based classification methods. Finally, most of the studies involving deep learning for mammography images have been performed using datasets from the same sources, both geographically and equipment-wise. One of the strengths of our method is that we implemented the proposed model using datasets from three different sources as well as with the mixed dataset from these three sources acquired from different locations using different equipment.

This study had some limitations. Firstly, it did not include a reader study to compare the performance of patchless deep-transfer learning against that of radiologists. The reason for this is that the nature of the used datasets does not allow us to do so. We are currently trying to obtain other datasets that are private and suitable for reader study in order to compare the performance of our patchless multi-stage transfer learning model against the performance of radiologists. This would help us to confidently conclude the extent of the performance of our deep-transfer learning model. Additionally, the ethnicity ratio within each dataset is not proportional for white and black people because of the origins of the datasets. A large portion of the datasets were of white people. Therefore, it is obvious that any model trained on such a dataset would be biased against under-represented data sources, and we doubt that our model will also be susceptible to such outlays. Therefore, a dataset that represents all ethnicities equally should be used to ensure that this model is unbiased for every ethnicity. The other limitation of the work is that we used only image information to retrieve the results of diagnosis, whereas radiologists utilize both image information and other patient information for diagnosis. A recent study involving patient information proved that the performance of deep learning can be improved with the use of patient information in addition to image information. Further investigation involving the use of patient information in addition to image information in our model could result in an improved performance of the proposed patchless multi-stage transfer-learning model. Furthermore, we did not study the effect of using different dataset sizes for cancer cell line images. The effect of increasing or decreasing the dataset size has to be investigated in the future to determine how the performance of deep-transfer learning would be affected. Moreover, we used cervical, lung, and breast cancer cell lines. We did not study the effect of using additional types of cancer cell lines or only breast cancer cell lines. Therefore, investigations considering the use of additional cancer cell lines should be carried out to fully understand and describe the effects of the use of cancer cell lines on the performance of the proposed deep-transfer learning model. We attempted to investigate the robustness of the hypothesis of patchless deep-transfer learning using two additional CNNs on top of the EfficientNetB2 model, including InceptionV3 and ResNet50 models; as well as two additional optimizers (Adam and SGD). It is important to employ a patchless deep-transfer learning model using additional CNN models and optimizers to investigate the performance patterns for different models and optimizers.

## 6. Conclusions

In conclusion, we developed a patchless multi-stage transfer learning model to distinguish between benign and malignant mammographic breast mass images. To this end, we utilized multi-stage transfer learning in which an EfficientNetB2 model pre-trained on ImageNet was fine-tuned via transfer learning for three-class classification on a cancer cell line dataset. Then, this model was used as a pre-trained model to classify mammographic breast mass images as benign or malignant. We recorded better performance on Digital Database for the Screening Mammography (DDSM) dataset, INbreast dataset, Mammographic Image Analysis Society (MIAS) dataset, and the mixture of these three datasets using our patchless deep-transfer learning model compared to the conventional ImageNet-based transfer learning and whole image and patch classifier model. Moreover, the proposed model demonstrated better performance than that of previous studies on the same datasets. This study has also improved computational efficiency by alleviating the need for patch separation to obtain a region of interest before classification, which has been used in previous studies to achieve high performance. The proposed method is important for solving the need for a large training dataset, for decreasing the computational burden in training, and for implementing mammography image-based deep-learning models. With improvements involving further studies, this work could be used as a tool to assist radiologists in the early diagnosis of breast cancer via mammography.

## Figures and Tables

**Table 1:** Mammogram datasets summary.

Characteristics	DDSM	INbreast	MIAS
Origin	USA	Portugal	UK
Age	Yes	Yes	No
Number of cases	2620	115	161
Views	MLO and CC	MLO and CC	MLO
Number of images	10,480	410	322
Resolution	8 and 16 bits/pixel	14 bits/pixel	8 bits/pixel
Benign: malignant ratio	0.65:0.35	0.72:0.28	0.84:0.16
Lesion type	All types of lesions	All types of lesions	All types of lesions
Annotation	Pixel level annotation	Annotation including label of individual finding	Center and ROI
Breast density information	Yes	Yes	Yes

DDSM: Digital Database for Screening Mammography, MIAS: Mammographic Image Analysis Society, USA: United States of America, UK: United Kingdom, MLO: mediolateral oblique, CC: craniocaudal, ROI: region of interest.

**Table 2:** Dataset categories.

Dataset	Category	Sub-Category	Dataset Size	Validation	Test
DDSM	Benign	-	3582	1194	1194
	Malignant	-	4293	1431	1431
INbreast	Benign	-	1512	504	504
	Malignant	-	3066	1022	1022
MIAS	Benign	-	1422	474	474
	Malignant	-	864	288	288
Mixed	Benign	DDSM	3582	1194	1194
		INbreast	1512	504	504
		MIAS	1422	474	474
		Total	6516	2172	2172
	Malignant	DDSM	4293	1431	1431
		INbreast	3066	1022	1022
		MIAS	864	288	288
		Total	8233	2741	2741

DDSM: Digital Database for Screening Mammography, MIAS: Mammographic Image Analysis Society.

**Table 3:** Model additional layers size.

Layer Type	Input	Output
Input Layer	16 × 227 × 227 × 3	16 × 227 × 227 × 3
EfficientNetB2	Load EfficientNetB2 from Keras and remove classifier & input Layer	
Global Average Pooling	16 × 7 × 7 × 1408	16 × 1408
Fully Connected Layer1 with L2	16 × 1408	16 × 1024
Fully Connected Layer2	16 × 1024	16 × 8
Fully Connected Layer3	16 × 8	16 × 8
Softmax	16 × 8	16 × 2

**Table 4:** Results of learning using early stop and fixed epoch.

Dataset	Training Condition	Validation Accuracy	Loss	Stopping Epoch
DDSM	Early stop with patience = 5	100	0.65	150
	Early stop with patience = 5	99.97	0.64	150
	Fixed epoch of 150	100	0.05	150
INbreast	Early stop with patience = 5	99.93	0.078	150
	Early stop with patience = 5	99.93	0.08	150
	Fixed epoch of 150	99.93	0.078	150
MIAS	Early stop with patience = 5	99.92	0.87	150
	Early stop with patience = 5	99.92	0.87	150
	Fixed epoch of 150	99.92	0.86	150
Mixed dataset	Early stop with patience = 5	99.95	0.05	133
	Early stop with patience = 5	99.98	0.07	150
	Fixed epoch of 150	99.98	0.07	150

**Table 5:** Performance summary of patchless multi-stage transfer learning based on EfficientNetB2 architecture with Adagrad optimizer.

Dataset	F1	AUC	Test Accuracy	Sensitivity	Specificity
DDSM	1	1	1	1	1
INbreast	0.9995	0.9994	0.9993	0.9996	0.9992
MIAS	0.9989	0.9993	0.9992	0.9987	1
Mixed	0.9998	0.9998	0.9998	1	0.9997

AUC: area under receiver operating curve; DDSM: Digital Database for Screening Mammography; MIAS: Mammographic Image Analysis Society database.

**Table 6:** Results of robustness analysis of the proposed system across different CNN models and optimizers.

Dataset	CNN-Optimizer Combination	F1-Score	AUC	Test Accuracy	Sensitivity	Specificity
DDSM	EfficientNetB2-Adagrad	1.0	1	1.0	1.0	1.0
	EfficientNetB2-Adam	0.99993	0.99993	0.99992	1.0	0.99986
	EfficientNetB2-SGD	1.0	1.0	1.0	1.0	1.0
	ResNet50-Adagrad	1.0	1.0	1.0	1.0	1.0
	ResNet50-Adam	0.94108	0.89991	0.90898	0.79983	1.0
	ResNet50-SGD	0.99986	0.99986	0.99984	1.0	0.99972
	InceptionV3-Adagrad	1.0	1.0	1.0	1.0	1.0
	InceptionV3-Adam	0.88227	0.8	0.81809	0.6	1.0
	InceptionV3-SGD	1.0	1.0	1.0	1.0	1.0
INbreast	EfficientNetB2-Adagrad	0.99951	0.99941	0.99934	0.99960	0.99921
	EfficientNetB2-Adam	0.99872	0.99802	0.99829	0.99722	0.99882
	EfficientNetB2-SGD	0.99664	0.99637	0.99554	0.99880	0.99393
	ResNet50-Adagrad	0.99892	0.99821	0.99855	0.99722	0.99921
	ResNet50-Adam	0.97055	0.96968	0.96371	0.98730	0.95209
	ResNet50-SGD	0.99647	0.99486	0.99528	0.99365	0.99608
	InceptionV3-Adagrad	0.99793	0.99764	0.99724	0.99880	0.99647
	InceptionV3-Adam	0.99786	0.99603	0.99711	0.99285	0.99921
	InceptionV3-SGD	0.99892	0.99852	0.99855	0.99841	0.99863
MIAS	EfficientNetB2-Adagrad	0.99896	0.99936	0.99921	0.99873	1.0
	EfficientNetB2-Adam	0.99860	0.99874	0.99895	0.99957	0.99791
	EfficientNetB2-SGD	0.99310	0.99564	0.99475	0.99199	0.99930
	ResNet50-Adagrad	0.99193	0.99242	0.99396	0.99873	0.98611
	ResNet50-Adam	0.95908	0.96780	0.96825	0.96962	0.96597
	ResNet50-SGD	0.99235	0.99365	0.99422	0.99536	0.99236
	InceptionV3-Adagrad	0.99614	0.99645	0.99711	0.99915	0.99375
	InceptionV3-Adam	0.99450	0.99608	0.99580	0.99494	0.99722
	InceptionV3-SGD	0.99476	0.99554	0.99606	0.99831	0.99236
Mixed	EfficientNetB2-Adagrad	0.99985	0.99985	0.99983	1.0	0.99970
	EfficientNetB2-Adam	0.99919	0.99913	0.99910	0.99935	0.99890
	EfficientNetB2-SGD	0.99926	0.99926	0.99918	0.99990	0.99861
	ResNet50-Adagrad	0.99905	0.99893	0.99894	0.99889	0.99897
	ResNet50-Adam	0.93016	0.88472	0.89688	0.77956	0.98986
	ResNet50-SGD	0.99766	0.99737	0.99739	0.99714	0.99759
	InceptionV3-Adagrad	0.99828	0.99806	0.99808	0.99788	0.99824
	InceptionV3-Adam	0.88094	0.79390	0.81700	0.59410	0.99365
	InceptionV3-SGD	0.99821	0.99797	0.99800	0.99769	0.99824

SGD: stochastic gradient descent; CNN: convolutional neural network; AUC: area under receiver operating curve; DDSM: Digital Database for Screening Mammography; MIAS: Mammographic Image Analysis Society database.

**Table 7:** Comparison of the proposed multistage transfer learning against conventional transfer learning.

Model	Dataset Type	CNN Architecture	Optimizer	Time (h)	Five-Fold Cross Validation Test Accuracy (%)
Best practice Conventional TL	DDSM	ResNet50	Adam	1.846567529	85.723
	INbreast	ResNet50	Adam	1.824081421	83.566
	MIAS	ResNet50	Adam	1.805489539	90.670
	Mixed	ResNet50	Adam	1.858144065	86.335
Multistage TL with the same set up as CTL	DDSM	ResNet50	Adam	1.711060605	90.898
	INbreast	ResNet50	Adam	1.708678728	96.371
	MIAS	ResNet50	Adam	1.694282732	96.825
	Mixed	ResNet50	Adam	1.724357648	89.688
Multistage TL with our best model	DDSM	EfficientNetB2	Adagrad	1.60336038	100
	INbreast	EfficientNetB2	Adagrad	1.51702123	99.934
	MIAS	EfficientNetB2	Adagrad	1.50130263	99.921
	Mixed	EfficientNetB2	Adagrad	1.62434423	99.983

CNN: convolutional neural network; TL: transfer learning; CTL: conventional transfer learning; DDSM: Digital Database for Screening Mammography; MIAS: Mammographic Image Analysis Society database; hr.: hour.

**Table 8:** Comparison of the proposed patchless multistage transfer learning method against the patch and whole-image classifier.

Fold Number	Patch and Whole Image Classifier		Proposed Patchless Multistage Transfer Learning Method	
	Accuracy (%)	Time (h)	Accuracy (%)	Time (h)
Fold 1	98.165	2.1730723	99.213	1.714756812
Fold 2	77.129	2.12583438	99.737	1.757123122
Fold 3	87.614	2.10610313	99.344	1.736005956
Fold 4	94.695	2.09312669	99.279	1.730858592
Fold 5	99.476	1.71885766	99.017	1.733659677
Average	91.416	2.043398834	99.344	1.734480832

**Table 9:** Comparison of the proposed multistage transfer learning method with the state-of-the-art mammographic breast cancer classification methods.

Paper	Application	Image Dataset	Dataset Size	Model Validation	CNN Model	AUC	Accuracy (%)
Al-masni et al. [22]	Classification	DDSM	600 with augmentation	5-fold CV	CNN, F-CNN	0.9645	96.33
Al-antari et al. [21]	Classification	DDSM, INbreast	9240 DDSM and 2266 INbreast with augmentation	5-fold CV	CNN, ResNet50, InceptionResNet-V2	CNN = 0.945, ResNet-50 = 0.9583, and InceptionResNet-V2 = 0.975 on DDSM and CNN = 0.8767, ResNet50 = 0.9233, and InceptionResNet-V2 = 0.9391 on INbreast	CNN = 94.5, ResNet-50 = 95.83, and InceptionResNet-V2 = 97.5 on DDSM and CNN = 88.74, ResNet50 = 92.55, and InceptionResNet-V2 = 95.32 on INbreast
Ribli et al. [63]	Classification	DDSM, SUD, INbreast	2949 with augmentation	NA	Faster RCNN	0.95	NA
Chougrad et al. [35]	Classification	DDSM, BCDR, INbreast, mixed, MIAS	6116 with augmentation	5-fold CV	Deep CNN	0.98 on DDSM, on 0.96 BCDR, 0.97 on INbreast, and 0.99 on MIAS	97.35 on DDSM, on 96.67 BCDR, 95.50 on INbreast, and 98.23 on MIAS
Lotter et al. [14]	Classification	DDSM	10,480 with augmentation	CV by patient	Wide ResNet	0.92	NA
Dhungel et al. [38]	Classification	INbreast	410 without augmentation	5-fold CV	CNN, RF, BO	0.69–0.76 MUI, 0.8–0.91 MS	Maximum of 95%
Saraswathi & Srinivasan [64]	Classification	MIAS	322 without augmentation	10-fold CV	FCRN	NA	94.7
The proposed method	Classification	DDSM, INbreast, MIAS, mixed	13,128 DDSM, 7632 INbreast, and 3816 MIAS. 24,576 mixed	5-fold CV	EfficientNetB2	1 on DDSM, 0.9995 on INbreast, 0.9989 on MIAS, and 0.9998 on mixed dataset	100 on DDSM, 99.93 on INbreast, 99.92 on MIAS, and 99.98 on mixed dataset

CNN: Convolutional Neural Network; AUC: area under receiver operating curve; CV: cross-validation; DDSM: Digital Database for Screening Mammography; SUD: Semmelweis University dataset; F-CNN: Fourier Convolutional Neural Networks; NA: not available; Faster RCNN: faster region-based convolutional neural network; BC: breast cancer; BCDR: breast cancer digital repository; MIAS: Mammographic Image Analysis Society database; DM: digital mammograms; DCNN: Deep Convolutional Neural Network; MIAS: Mammographic Image Analysis Society database; RF: Random Forests; MUI: minimal user intervention; MS: manual set-up; BO: Bayesian optimization; FCRN: Fully complex-valued relaxation neural network.

## Data Availability

In this study, we used publicly available mammogram images, namely, Digital Database for Screening Mammography (DDSM) dataset (accessible at http://www.eng.usf.edu/cvprg/mammography/database.html (accessed on 8 September 2021)), INbreast dataset accessible at https://biokeanos.com/source/INBreast (accessed on 8 September 2021), and Mammographic Image Analysis Society (MIAS) dataset (accessible at http://peipa.essex.ac.uk/info/mias.html (accessed on 8 September 2021)). The Biomedical imaging, therapeutics, and sensing lab (BITS) cancer cell line image dataset can be made available for reasonable requests by contacting the corresponding authors.
